# Regularity of kinematic data between single and dual-task treadmill walking in people with Parkinson’s disease

**DOI:** 10.1186/s12984-021-00807-5

**Published:** 2021-02-01

**Authors:** Samira Ahmadi, Tarique Siragy, Julie Nantel

**Affiliations:** School of Human Kinetics, University of Ottawa, Ottawa, ON Canada

**Keywords:** Sample entropy, Complexity index, Time delay, Gait, Dual-task, Parkinson’s disease

## Abstract

**Background:**

Regularity, quantified by sample entropy (SampEn), has been extensively used as a gait stability measure. Yet, there is no consensus on the calculation process and variant approaches, e.g. single-scale SampEn with and without incorporating a time delay greater than one, multiscale SampEn, and complexity index, have been used to calculate the regularity of kinematic or kinetic signals. The aim of the present study was to test the discriminatory performance of the abovementioned approaches during single and dual-task walking in people with Parkinson’s disease (PD).

**Methods:**

Seventeen individuals with PD were included in this study. Participants completed two walking trials that included single and dual-task conditions. The secondary task was word searching with twelve words randomly appearing in the participants’ visual field. Trunk linear acceleration at sternum level, linear acceleration of the center of gravity, and angular velocity of feet, shanks, and thighs, each in three planes of motion were collected. The regularity of signals was computed using approaches mentioned above for single and dual-task conditions.

**Results:**

Incorporating a time delay greater than one and considering multiple scales helped better distinguish between single and dual-task walking. For all signals, the complexity index, defined as the summary of multiscale SampEn analysis, was the most efficient discriminatory index between single-task walking and dual-tasking in people with Parkinson's disease. Specifically, the complexity index of the trunk linear acceleration of the center of gravity distinguished between the two walking conditions in all three planes of motion.

**Conclusions:**

The significant results observed across the 24 signals studied in this study are illustrative examples of the complexity index’s potential as a gait feature for classifying different walking conditions.

## Background

Researchers have proposed and studied a plethora of measures to quantitatively analyze walking [1]. Among them, entropy measures, such as Sample Entropy (SampEn), have shown great potential to successfully discern interrupted gait from uninterrupted [2]. Entropy measures represent regularity or predictability of signals and have been shown to distinguish between fallers and non-fallers [3], single and dual-task walking conditions [2], patients with various neurological and/or physical impairments and control groups [4]. Although first calculated for discrete stride-interval time series [5], entropy measures are increasingly applied to continuous human signals [3, 4, 6–9]. Trunk linear acceleration [4, 8, 10–13], lower extremity’s angular velocity [10, 13], and center of foot pressure [6] have been used with various entropy measures. Unlike inter-stride signals, such as step time, kinematic signals offer important information on motor control strategies as they comprise intra-stride details of gait cycles [6]. A previous study suggests that the within-stride phases, such as  the weight transfer phase, are critical for gait stability [14]. These phases are actively engaged with intra-stride patterns of cortical activity [15] which are involved in steady-speed human locomotion. [15, 16].

SampEn, among different types of entropy measures, is the most commonly used measure for human gait analysis. This single-scale regularity measure is representative of difficultness of describing the patterns of the trajectory of a system [17]. Single-scale analysis, however, is not capable of representing the multiscale richness of information embedded in a signal. Therefore, multiscale sample entropy (MSE) [5], which quantifies regularity on multiple scales, was introduced. One limitation of MSE is that it is reported as multiple SampEn values over a range of scales and does not produce a single value. This limitation hinders its application in gait analysis, especially when single quantitative features are rather desirable. Complexity index (CI) [18] calculated as the sum of SampEn values over a range of scales was introduced as a solution. Both single-scale and multiscale SampEn have shown great potential to capture the increased risk of fall despite not having incorporated a time-delay greater than one [2, 8]. For time series generated by nonlinear dynamics that have a long range correlation, it has been shown that using a higher time delay can provide additional information regarding the characteristics of a time series when conducting comparative analyses [19, 20]. In finite noisy time-series data, an appropriate choice of time delay [21] is necessary to properly reconstruct the state space as a too small time delay would produce a compressed attractor along the identity line [22]. In other words, the time delay is chosen such that the components of an embedding window or a template (which are the building blocks of nonlinear measures) are independent [23]. Incorporating a non-unity time delay has been used to calculate other gait stability measures, such as largest Lyapunov exponents, when applied to continuous kinematic gait signals [24, 25]. Nonetheless, the influence of incorporating a non-unity time delay, i.e. a time delay greater than one, when studying continuous signals with entropy measures is yet to be determined. The three approaches mentioned earlier, i.e. single-scale SampEn with and without a time delay greater than one, multiscale SampEn, and complexity index when calculated for continuous signals could somehow yield different results.

As Parkinson’s disease (PD) is the second most common neurodegenerative disease worldwide, after Alzheimer’s Disease, and has an exacerbated fall risk during walking, PD provides an opportunity to examine the efficacy of the aforementioned entropy metrics [26–28]. Epidemiological evidence demonstrates that the incidence of PD ranges between 10 and 50 per 100,000 individuals and has a prevalence between 100 and 300 per 100,000 individuals [29, 30]. Additionally, the number of individuals diagnosed with PD is expected to double worldwide by 2030 [31]. PD is a progressive neurodegeneration of dopaminergic neurons within the Basal Ganglia [26, 27]. In addition to causing PD’s cardinal symptoms (bradykinesia, rigidity, and tremor), the Basal Ganglia’s reduced functionality impairs automatic motor processes that contribute to effective locomotion [1, 32–34]. To overcome the impaired automaticity of the Basal Ganglia, previous research proposes that individuals with PD attempt to recruit relatively intact higher-level cortical structures (pre-frontal and frontal cortices) to direct conscious attentional control on their locomotion [32, 35]. However, when attention is divided between simultaneous tasks, the attentional resources required for effective compensation of impaired neuromuscular pathways become strained [32, 35]. For instance, dual-tasking evidence demonstrates that during the presence of a concurrent mental task, there is a breakdown in the gait pattern for individuals with PD which increases their fall risk [32, 33, 35, 36]. Hillel et al. discussed that changes in PD gait performance during dual-tasks are more representative of how these individuals ambulate in their everyday lives where multitasking is ever present in constantly changing environments [37]. To-date, however, it remains unexamined whether incorporating a non-unity time delay and multiscale analysis on SampEn results affects the sensitivity of entropy to respond to dual-tasks.

The main objective of the current study was to investigate the effect of incorporating a non-unity time delay and multiscale analysis on SampEn results. It was hypothesized that these approaches would increase the ability of SampEn to discriminate between single and dual-task trials. In addition, another objective of this paper was to determine whether the SampEn of eight different signals, each in three directions, would result in the same outcome. These eight different signals are trunk linear acceleration at sternum level, linear acceleration of the center of gravity, and angular velocity of least-affected and most-affected feet, shanks, and thighs. It was hypothesized that the discriminatory ability of SampEn would be greater when calculated for signals in the mediolateral direction based on previous studies showing an increased demand on mediolateral stability during visual perturbations [38–40].

## Methods

### Participants

Twenty (13 males; 7 females) individuals with idiopathic PD (confirmed by a neurologist), aged between 48 and 79 years old (age: 63.8 ± 9.0 years, mass: 72.3 kg ± 19.4, height: 172.8 cm ± 8.0) were recruited from the Ottawa-Gatineau region. Due to incomplete data collection, only 17 participants (11 males and 6 females, 64.8 ± 7.4) were used in the analysis. Participants were assessed with the original Unified Parkinson’s Disease Rating Scale Motor examination (11 ± 6) and were between I-III on the Hoehn and Yahr scale. Average disease duration (8.0 ± 5.1 years) and age at onset (56.8 ± 9.6 years) data were collected. Further, seven participants reported freezing of gait based on the Freezing of Gait Questionnaire. Participants were tested on their optimally medicated state. Exclusion criteria encompassed if participants reported any physical discomfort using a virtual reality system, reported any injuries and/or orthopedic surgeries that could interfere with walking, could only ambulate with the assistance of a walking aid and had any additional conditions other than PD. All participants provided written informed consent and the study was approved by the University of Ottawa Research Ethics Boards (REB) and Ottawa Hospital Research Institute (OHRI) in accordance with the Helsinki protocol.

### Procedures

Participants completed two treadmill walking trials, where they walked on a straight path (i.e. the treadmill belt) with their preferred walking speed, that included single and dual-task conditions. These two trials were part of a larger protocol [41, 42]. The single-task trial had a duration of three minutes while the dual-task trial lasted two minutes. Prior to data collection, participants walked for approximately 20 s to achieve steady-state walking. The dual-task consisted of a word searching task with twelve words randomly appearing in the participants’ visual field. Words were projected one at a time on a screen directly in front of the participants. Words appeared randomly on either the left or right side of the screen at angles varying between 20 and 70 degrees. The duration of each word was 3 s with a 2–4 s pause between following words. Participants verbally called out the words as they appeared [41]. The word searching task was chosen as a more ecologically representation of a potential secondary task that individuals would encounter in their daily lives when walking. Specifically, this task reflects scanning the environment for relevant visual cues such as when passing by signs which provide direction.

Three-dimensional motion analysis was completed using the CAREN-Extended System (Motek Medical, Amsterdam NL). This system combines a six degrees-of-freedom motion platform with an embedded dual-belt treadmill, 12 camera Vicon motion capture system, 180-degree projector screen, and a safety harness. A 57-marker set for tracking full body kinematics was used. Kinematic data were collected at 100 Hz.

### Regularity measures

We briefly describe SampEn and define its single-scale and multiscale implementation with and without a non-unity time delay. Next, complexity index as a summarizing index of MSE is explained.

Consider a time series of length $N$ given below:

1 $$X=\left\{x\left(1\right),x\left(2\right),\dots ,x\left(N\right)\right\} or X=\left\{x\left(j\right):1\le j\le N\right\}$$

The value for template size ($m$) is chosen to construct series of pairs, size $m$ as:

2 $${X}_{m}\left(i\right)=\left\{x\left(i+k\right):0\le k\le m-1\right\}, 1\le i\le N-m+1$$

Next, matching templates are found by comparing their Chebyshev distance (denoted as $\mathrm{d}\left|.\right|$) to a pre-determined threshold size ($r$) while excluding self-comparison. Next, the variable ${B}_{i}$, which is the number of pairs satisfying the aforementioned criteria, is built.

3 $${B}_{i}^{m}\left(\mathrm{r}\right)=\frac{1}{N-m-1} \left(\#\mathrm{ of d}\left|{X}_{m}\left(i\right)-{X}_{m}\left(j\right)\right| \le r, where j=1:N-m \& i\ne j\right)$$

4 $$\mathrm{d}\left|{X}_{m}\left(i\right)-{X}_{m}\left(j\right)\right|=\mathrm{max}\left\{\left|x\left(i+k\right)-x\left(j+k\right)\right|:0\le k\le m-1\right\}$$

Next, ${B}^{m}\left(r\right)$ is defined as:

5 $${B}^{m}\left(r\right)=\frac{1}{N-m}{\sum }_{i=1}^{N-m}{B}_{i}^{m}\left(r\right)$$

This process is repeated for $m+1$ and $r$ to form ${A}^{m}\left(r\right)$:

6 $${A}_{i}^{m}\left(\mathrm{r}\right)=\frac{1}{N-m-1} (\#\mathrm{ of d}\left|{X}_{m+1}\left(i\right)-{X}_{m+1}\left(j\right)\right|\le r), \mathrm{where} j=1:N-m \& i\ne j$$

7 $${A}^{m}\left(r\right)=\frac{1}{N-m}{\sum }_{i=1}^{N-m}{A}_{i}^{m}\left(r\right)$$

Lastly, single-scale SampEn [43] is calculated based on ${B}^{m}\left(r\right)$ and ${A}^{m}\left(r\right)$ as

8 $$\mathrm{SampEn}\left(m,r,N\right)= -\mathrm{ln}\frac{{A}^{m}\left(r\right)}{{B}^{m}\left(r\right)}$$

where $m$, $r$ and $N$ are the template size (i.e., the length of template vector), tolerance size and the length of time series, respectively [6].

For MSE, consecutive coarse-grained time series are constructed by averaging a successively increasing number of data points in non-overlapping windows. The number of data points in each window is determined by the scale factor which ranges from 1 to 20 in this study. MSE is usually presented as SampEn for each one of the coarse-grained time series plotted as a function of scale factor. And the complexity index is defined as the sum of the SampEn values of different scales [18].

In order to incorporate a non-unity time delay, a time delay $(\tau )$, which has been calculated using the minimum average mutual information method [22], is used when constructing the templates in [2] as below while the rest of the calculation process remain the same.

9 $${X}_{m}\left(i\right)=\left\{x\left(i+k\tau \right):0\le k\le m-1\right\}, 1\le i\le N-m+1$$

### Data processing

**Fig. 1:**
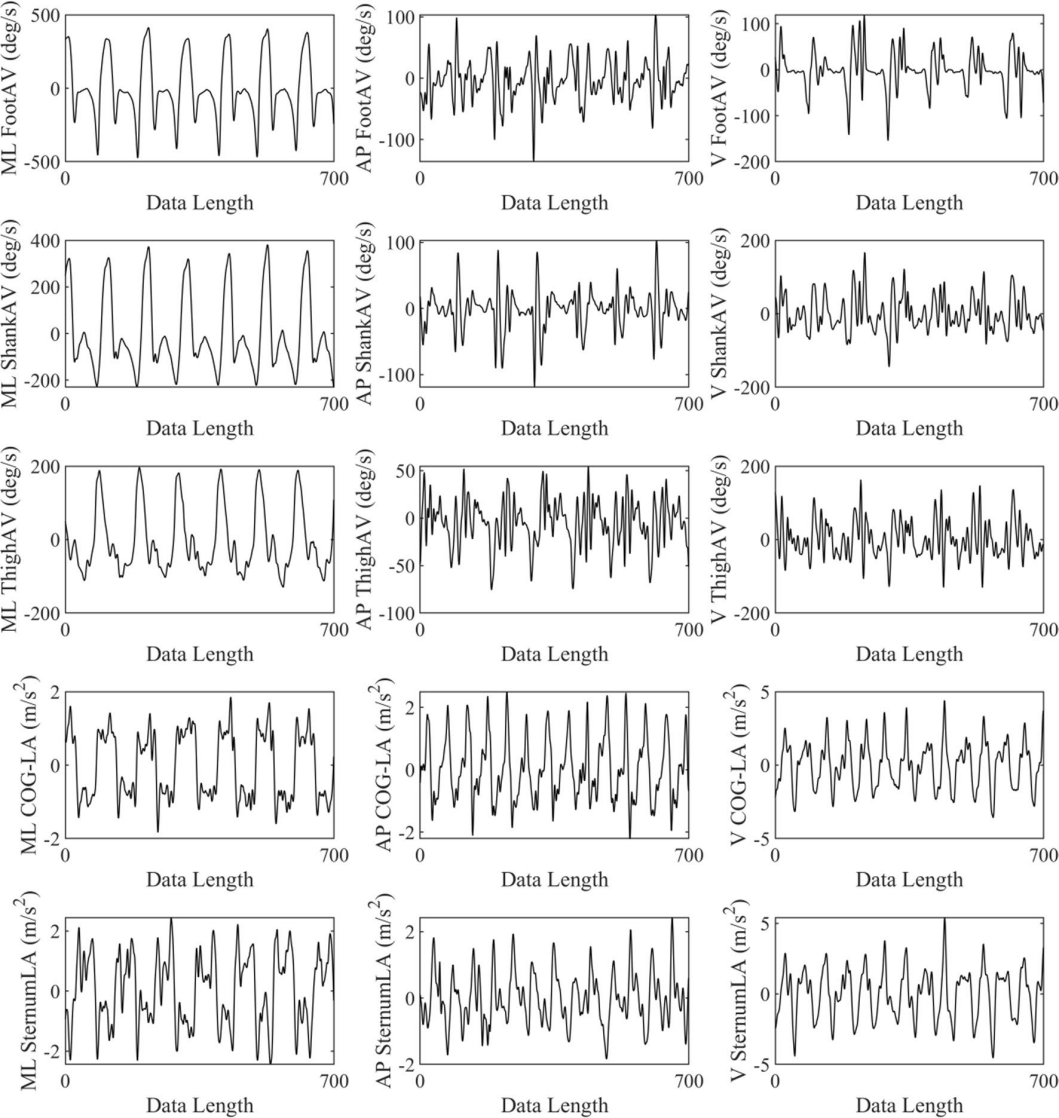
A 7-s segment of biological data for continuous foot angular velocity (FootAV), shank angular velocity (ShankAV), thigh angular velocity (ThighAV), COG linear acceleration (COG-LA), and sternum linear acceleration (SternumLA) in ML (left), AP (middle) and V (right) directions

Markers data were processed in Vicon Nexus (Nexus 2.6, Oxford, UK) and 3D kinematic calculations were performed in Visual 3D. A 4th order, low-pass Butterworth filter with a 10 Hz cut-off frequency was used to filter marker data. Visual 3D calculates the center of gravity based on the anthropometric data provided by Hanavan [44]. Center of gravity velocity and acceleration were calculated as the first and second derivative of the center of gravity’s position. For this study, only trunk linear acceleration at sternum level (SternumLA), center of gravity linear acceleration (COG-LA), foot angular velocity (FootAV), shank angular velocity (ShankAV), and thigh angular velocity (ThighAV), each in mediolateral (ML), anteroposterior (AP), and vertical (V) directions were exported from Visual 3D (see Fig. 1). Subsequently, the exported data were analyzed in MATLAB R2019b. For angular velocity signals, results are reported for the most affected (MA) and the least affected (LA) foot, shank, and thigh instead of left and right categorization.

70 strides of each signal were resampled to have the same $108\times 70$ data points, where 108 was the average number of data points per stride for 17 participants. This data manipulation was recommended to account for variant gait speed among participants and across trials [6]. Next, SampEn was calculated for each signal using a template size of 4, a tolerance size of 0.2 times the standard deviation of all time-series, and a time delay of one. Similarly, SampEn was recalculated for all signals using a template size of 4, a tolerance size of 0.2 times the standard deviation of all time-series, and a specified non-unity time delay. A systematic parameter selection [6] was followed to select template size and tolerance size, and the minimum average mutual information method was used to calculate the time delay [22]. A range of time delay values was obtained for each signal when calculated for different participants [45–47], and the median values were selected for the analysis (see Table 1). The dominant frequency of intrinsically periodic human gait signals is different across body segments, and it affects the calculated time delay [47]. Signals with less stride-to-stride changes have larger time delay values; i.e. fewer data points are needed to accurately reconstruct the state-space using a single time-series data [22].

**Table 1:** Median time delay in mediolateral, anteroposterior, and vertical directions

	ML	AP	V
FootAV	22	10	10
ShankAV	12	7	6
ThighAV	16	7	6
COG-LA	21	7	10
SternumLA	7	9	10

Subsequently, consecutive coarse-grained signals were constructed by averaging a successively increasing number of data points (up to 20) in non-overlapping windows. Next, SampEn was calculated for each coarse-grained signal using a template size of 4, a tolerance size of 0.2 times the standard deviation of all time-series of the same body segment, and a specified time delay greater than one. Time delay at each scale was the quotient of the division of the time delay at scale 1 by the scale values. For quotient values below 2, a time delay of 2 was chosen. This approach to time delay selection was developed after calculating the time delay for select signals using the minimum average mutual information method. Finally, the complexity index was computed by summing up SampEn values over scales 1 to 20.

### Statistical analysis

The normality of all dependent variables was checked using the Shapiro–Wilk normality test. Paired t-test and Wilcoxon signed ranks test were used respectively for variables normally distributed and non-normally distributed to investigate the effect of dual-tasking. A *p*-value less than 0.05 was considered significant in all tests.

## Results

For single-scale SampEn with a time delay of one (see Table 2), there was no significant change from single-task to dual-task walking conditions, except for vertical MA-FootAV (*Z* = − 2.580, *p* = 0.010) where SampEn increased significantly. For single-scale SampEn with a larger time delay (see Table 3), dual-tasking elicited a statistically significant change in LA-ThighAV in AP direction (*t* = − 3.898, *p* = 0.001)**,** MA-ShankAV in AP direction (*t* = − 3.031, *p* = 0.008), COG-LA in ML direction (*t* = − 2.818, *p* = 0.012), and SternumLA in ML (*t* = − 2.574, p = 0.020) and V direction (*t* = − 2.767, *p* = 0.014).

**Table 2:** Descriptive results (mean ± SD) and pairwise comparisons of the SampEn (with a time delay of one) of single-task and dual-task trials

Signal	Direction	Single-task	Dual-task	*t* or *Z*	*p*-value	DTI (%)
LA-FootAV	ML	0.064 ± 0.007	0.065 ± 0.007	− 1.007	0.329	1.4
	AP	0.332 ± 0.104	0.325 ± 0.103	− 0.544	0.586	− 2.0
	V	0.119 ± 0.044	0.125 ± 0.043	− 1.586	0.113	4.8
LA-ShankAV	ML	0.117 ± 0.022	0.117 ± 0.018	− 0.002	0.999	0.0
	AP	0.314 ± 0.087	0.311 ± 0.059	0.132	0.897	− 0.7
	V	0.389 ± 0.076	0.384 ± 0.070	0.613	0.549	− 1.3
LA-ThighAV	ML	0.165 ± 0.035	0.168 ± 0.035	− 0.689	0.501	2.0
	AP	0.424 ± 0.068	0.431 ± 0.069	− 0.667	0.514	1.8
	V	0.486 ± 0.063	0.480 ± 0.062	0.776	0.449	− 1.3
MA-FootAV	ML	0.063 ± 0.007	0.064 ± 0.009	− 1.076	0.298	2.3
	AP	0.271 ± 0.065	0.286 ± 0.068	− 1.475	0.160	5.5
	V	**0.099 ± 0.034**	**0.111 ± 0.040**	**− 2.580**	**0.010**	**11.6**
MA-ShankAV	ML	0.115 ± 0.021	0.117 ± 0.022	− 0.687	0.502	2.1
	AP	0.300 ± 0.055	0.307 ± 0.048	− 0.829	0.419	2.2
	V	0.360 ± 0.075	0.362 ± 0.066	− 0.239	0.814	0.4
MA-ThighAV	ML	0.164 ± 0.036	0.169 ± 0.045	− 1.087	0.293	3.4
	AP	0.409 ± 0.064	0.418 ± 0.050	− 0.834	0.417	2.3
	V	0.459 ± 0.062	0.460 ± 0.054	− 0.828	0.407	0.3
COG-LA	ML	0.323 ± 0.055	0.345 ± 0.054	− 1.617	0.125	6.5
	AP	0.450 ± 0.072	0.449 ± 0.059	0.114	0.911	− 0.2
	V	0.329 ± 0.041	0.329 ± 0.037	0.080	0.938	− 0.1
SternumLA	ML	0.363 ± 0.042	0.359 ± 0.048	0.710	0.488	− 1.1
	AP	0.474 ± 0.051	0.483 ± 0.058	− 1.065	0.287	2.0
	V	0.324 ± 0.052	0.330 ± 0.049	− 1.328	0.203	2.0

Values in bold indicate a significant (*p* < 0.05) difference. Dual-task interference (DTI) is calculated as DTI (%) = [(dual-task feature—single-task feature)/single-task feature] $\times$ 100

**Table 3:** Descriptive results (mean ± SD) and pairwise comparisons of the SampEn (with a non-unity time delay) of single-task and dual-task trials

Signal	Direction	Single-task	Dual-task	*t* or *Z*	*p*-value	DTI (%)
LA-FootAV	ML	0.572 ± 0.096	0.599 ± 0.111	− 1.112	0.266	4.8
	AP	1.296 ± 0.220	1.327 ± 0.202	− 1.633	0.102	2.4
	V	1.281 ± 0.340	1.313 ± 0.344	− 1.739	0.101	2.5
LA-ShankAV	ML	0.439 ± 0.054	0.455 ± 0.063	− 1.464	0.163	3.5
	AP	0.827 ± 0.204	0.840 ± 0.161	− 0.358	0.725	1.6
	V	1.165 ± 0.140	1.180 ± 0.147	− 0.902	0.380	1.3
LA-ThighAV	ML	0.544 ± 0.091	0.570 ± 0.123	− 1.642	0.120	5.0
	AP	**1.210 ± 0.154**	**1.303 ± 0.158**	**− 3.898**	**0.001**	**7.7**
	V	1.317 ± 0.185	1.350 ± 0.193	− 1.963	0.067	2.5
MA-FootAV	ML	0.539 ± 0.073	0.560 ± 0.105	− 1.325	0.204	3.8
	AP	1.174 ± 0.174	1.220 ± 0.186	− 1.561	0.138	4.0
	V	1.142 ± 0.286	1.193 ± 0.302	− 1.857	0.082	4.4
MA-ShankAV	ML	0.442 ± 0.049	0.455 ± 0.056	− 1.541	0.143	3.1
	AP	**0.825 ± 0.172**	**0.881 ± 0.201**	**− 3.031**	**0.008**	**6.8**
	V	1.108 ± 0.173	1.127 ± 0.162	− 1.228	0.237	1.8
MA-ThighAV	ML	0.511 ± 0.073	0.544 ± 0.098	− 1.662	0.116	6.6
	AP	1.178 ± 0.105	1.213 ± 0.102	− 1.108	0.284	2.9
	V	1.283 ± 0.161	1.321 ± 0.134	− 1.563	0.138	2.9
COG-LA	ML	**1.004 ± 0.139**	**1.061 ± 0.164**	**− 2.818**	**0.012**	**5.7**
	AP	1.090 ± 0.123	1.114 ± 0.120	− 2.015	0.061	2.2
	V	1.049 ± 0.139	1.067 ± 0.138	− 0.969	0.347	1.7
SternumLA	ML	**1.077 ± 0.143**	**1.122 ± 0.128**	**− 2.574**	**0.020**	**4.2**
	AP	1.405 ± 0.139	1.435 ± 0.157	− 1.121	0.279	2.1
	V	**0.992 ± 0.161**	**1.033 ± 0.182**	**− 2.767**	**0.014**	**4.1**

Values in bold indicate a significant (*p* < 0.05) difference. Dual-task interference (DTI) is calculated as DTI (%) = [(dual-task feature—single-task feature)/single-task feature] $\times$ 100

**Fig. 2:**
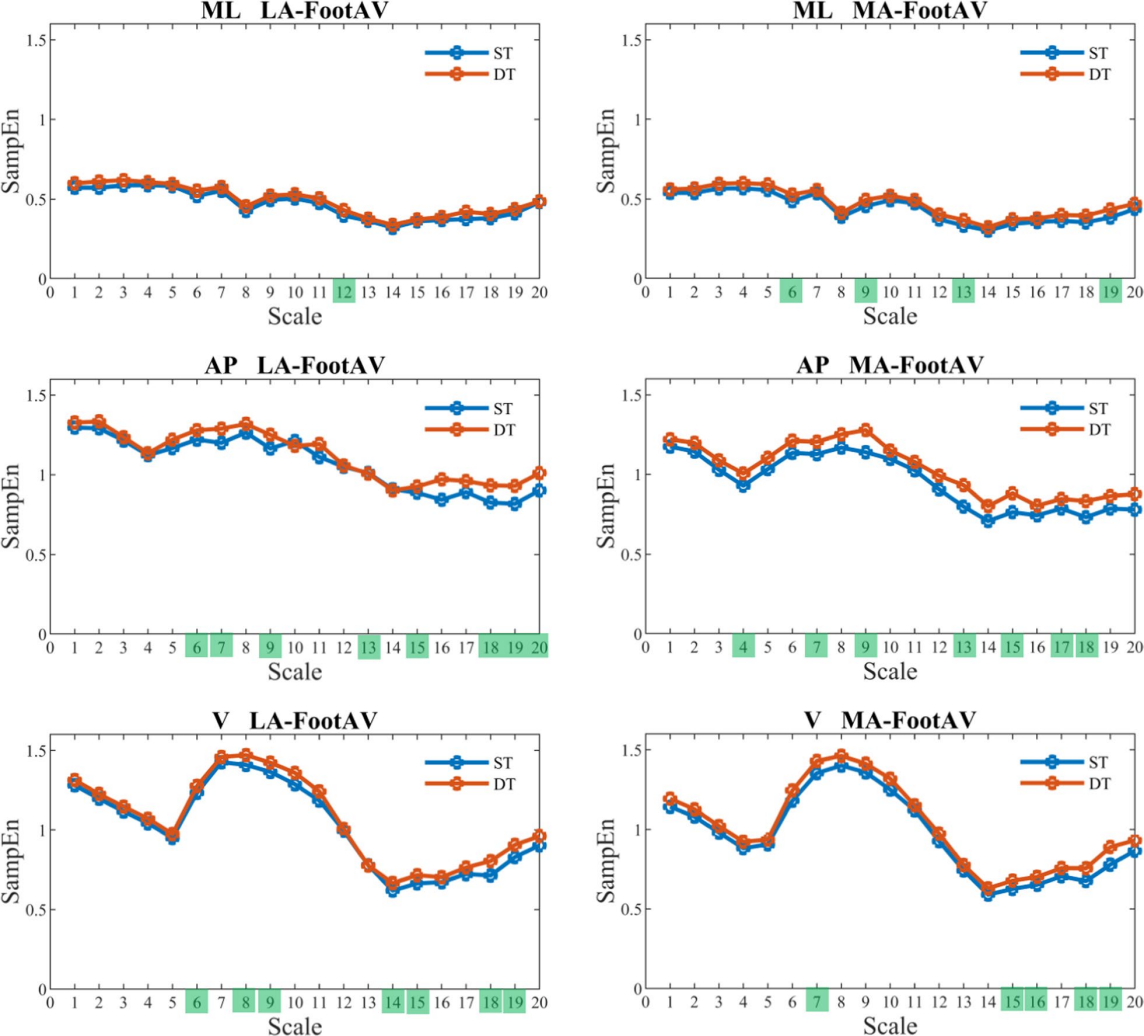
The SampEn of the least-affected (left column) and most-affected (right column) foot angular velocity (FootAV) in the ML (top row), AP (middle row), and V (bottom row) directions versus time scale for single-task (ST) and dual-task (DT) walking condition. Scales highlighted in green elicited significant increase (*p* < 0.05) in SampEn from ST to DT

**Fig. 3:**
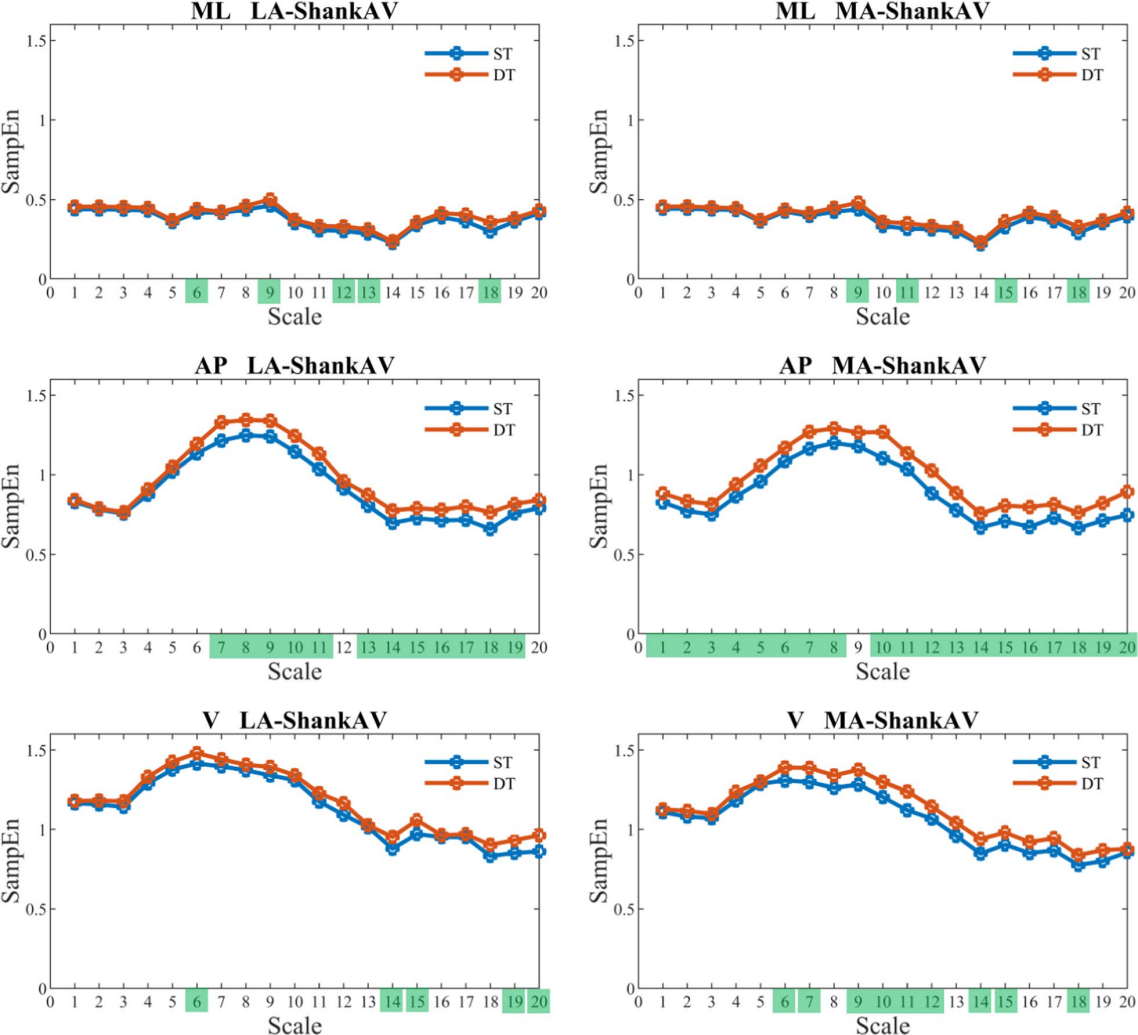
SampEn of the least-affected (left column) and most-affected (right column) shank angular velocity (ShankAV) in the ML (top row), AP (middle row), and V (bottom row) directions versus time scale for single-task (ST) and dual-task (DT) walking condition. Scales highlighted in green elicited significant increase (*p* < 0.05) in SampEn from ST to DT

**Fig. 4:**
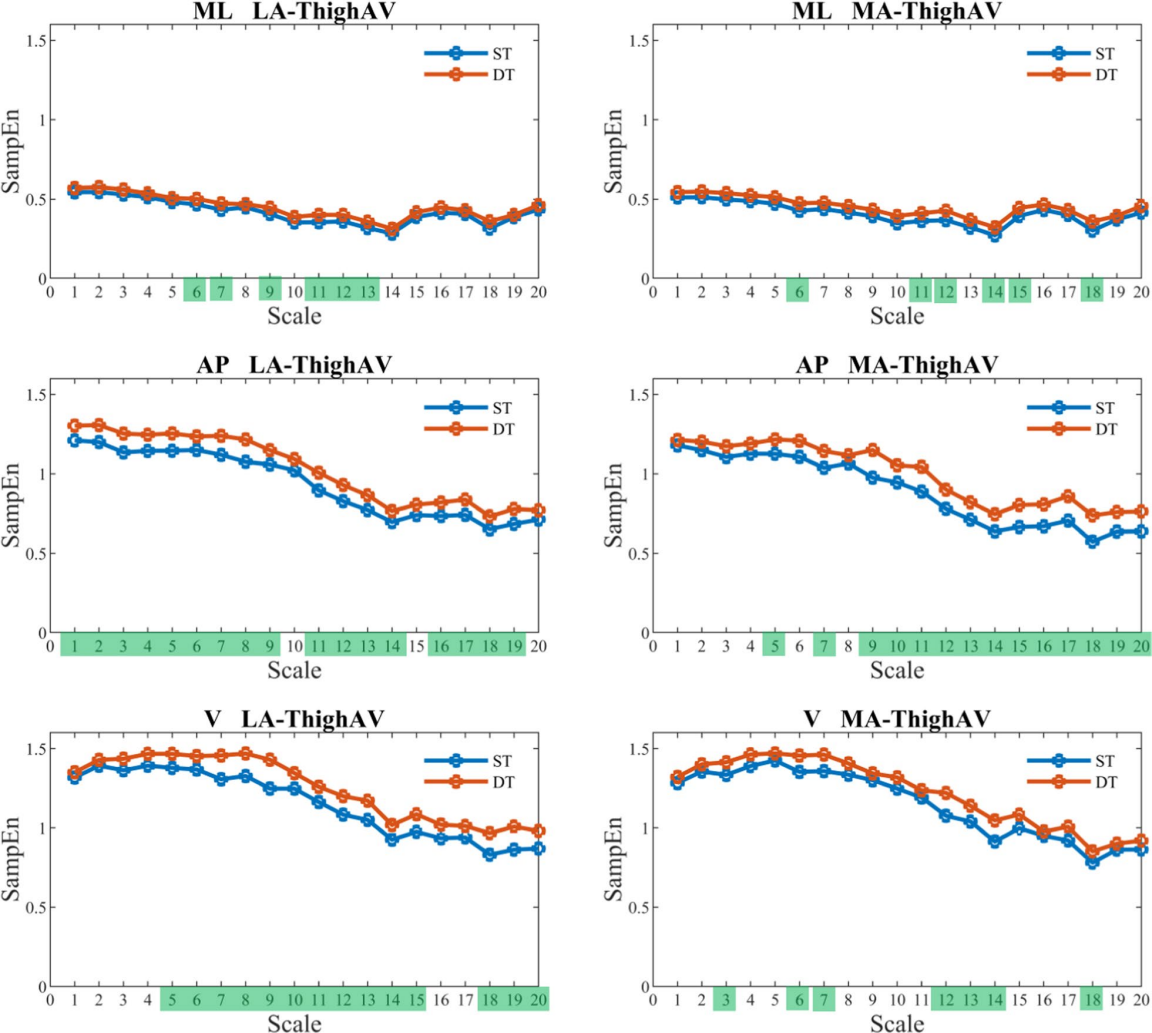
SampEn of the least-affected (left column) and most-affected (right column) thigh angular velocity (ThighAV) in the ML (top row), AP (middle row), and V (bottom row) directions versus time scale for single-task (ST) and dual-task (DT) walking condition. Scales highlighted in green elicited significant increase (*p* < 0.05) in SampEn from ST to DT

**Fig. 5:**
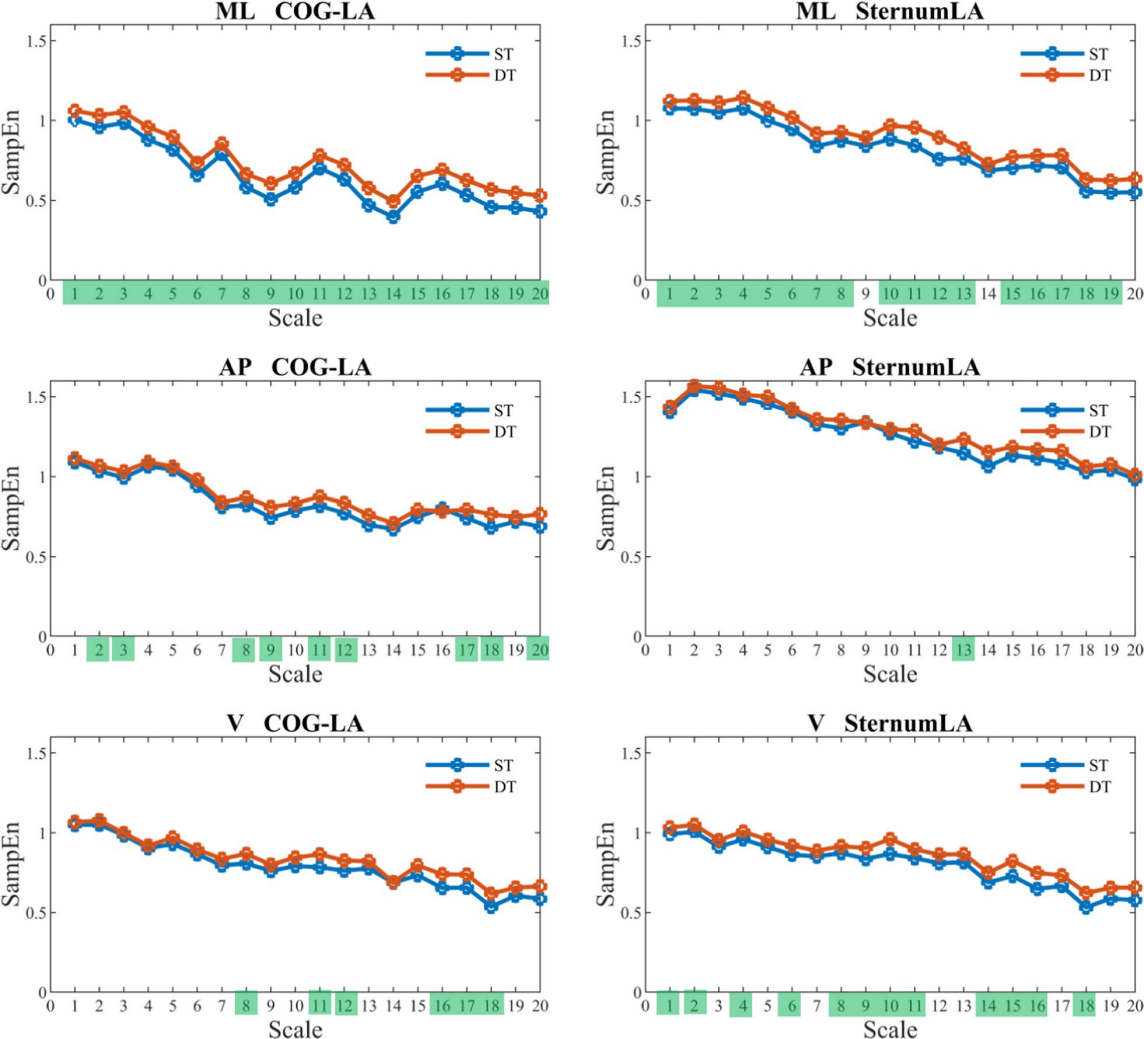
SampEn of the linear acceleration of COG (COG-LA) (left column) and linear acceleration of sternum (SternumLA) (right column) in the ML (top row), AP (middle row), and V (bottom row) directions versus time scale for single-task (ST) and dual-task (DT) walking condition. Scales highlighted in green elicited significant increase in SampEn from ST to DT

Figure 2, 3, 4, and 5 show that SampEn values vary with each scale. Nonetheless, the directional change from single-task to dual-task walking condition remains consistent for the examined signals. In other words, the SampEn of various signals tends to increase when participants engage in dual-tasking. A closer look reveals that LA-ShankAV and MA-ShankAV in AP direction (see Fig. 3), LA-ThighAV and MA-ThighAV in AP direction, LA-ThighAV in V direction (see Fig. 4), and COG-LA and SternumLA in ML direction (see Fig. 5) better distinguish between single-task and dual-task walking condition. These signals show significant changes across many time scales unlike the rest of the signals which discriminate between single and dual-task conditions only at sparse time scales.

Finally, the complexity index is presented in Table 4. The values for foot, shank, and thigh angular velocity in AP and V directions are significantly greater when dual-tasking. No significant changes were observed in the ML direction. For the COG-LA signal, the complexity index increased significantly in all three directions. SternumLA signal, however, was able to discriminate between the single and dual-task walking conditions in ML and V directions.

**Table 4:** Descriptive results (mean ± SD) and pairwise comparisons of the complexity index of single-task and dual-task trials

Signal	Direction	Single-task	Dual-task	*t* or *Z*	*p*-value	DTI (%)
LA-FootAV	ML	9.324 ± 1.318	9.826 ± 1.908	− 1.453	0.165	5.4
	AP	**21.384 ± 4.711**	**22.448 ± 3.953**	**− 1.965**	**0.049**	**5.0**
	V	**20.395 ± 3.098**	**21.265 ± 3.213**	**− 2.886**	**0.011**	**4.3**
LA-ShankAV	ML	7.470 ± 0.825	7.934 ± 1.258	− 1.804	0.090	6.2
	AP	**18.038 ± 2.215**	**19.323 ± 2.659**	**− 2.627**	**0.009**	**7.1**
	V	**22.550 ± 3.435**	**23.527 ± 3.774**	**− 2.634**	**0.018**	**4.3**
LA-ThighAV	ML	8.377 ± 1.458	9.015 ± 2.078	− 2.029	0.059	7.6
	AP	**18.705 ± 2.259**	**20.596 ± 2.918**	**− 3.747**	**0.002**	**10.1**
	V	**22.976 ± 3.500**	**25.023 ± 4.395**	**− 3.812**	**0.002**	**8.9**
MA-FootAV	ML	8.836 ± 1.002	9.455 ± 1.675	− 2.102	0.052	7.0
	AP	**18.989 ± 3.367**	**20.605 ± 3.826**	**− 2.293**	**0.036**	**8.5**
	V	**19.222 ± 2.755**	**20.317 ± 3.132**	**− 2.180**	**0.045**	**5.7**
MA-ShankAV	ML	7.390 ± 0.786	7.832 ± 1.221	− 1.999	0.063	6.0
	AP	**17.483 ± 3.013**	**19.470 ± 3.713**	**− 3.006**	**0.003**	**11.4**
	V	**21.143 ± 3.531**	**22.472 ± 3.594**	**− 2.420**	**0.028**	**6.3**
MA-ThighAV	ML	8.135 ± 1.250	8.987 ± 2.012	− 2.040	0.058	10.5
	AP	**17.713 ± 2.134**	**19.896 ± 2.858**	**− 3.123**	**0.007**	**12.3**
	V	**22.980 ± 3.272**	**24.439 ± 3.284**	**− 2.937**	**0.010**	**6.3**
COG-LA	ML	**12.996 ± 2.483**	**14.721 ± 3.245**	**− 4.662**	**0.000**	**13.3**
	AP	**16.645 ± 2.518**	**17.513 ± 3.237**	**− 2.372**	**0.031**	**5.2**
	V	**15.715 ± 3.267**	**16.680 ± 3.734**	**− 2.155**	**0.047**	**6.1**
SternumLA	ML	**16.497 ± 3.261**	**17.938 ± 3.689**	**− 3.820**	**0.002**	**8.7**
	AP	25.051 ± 3.567	25.877 ± 4.029	− 1.757	0.098	3.3
	V	**15.957 ± 3.536**	**17.195 ± 4.090**	**− 2.796**	**0.013**	**7.8**

alues in bold indicate a significant (*p* < 0.05) difference

Dual-task interference (DTI) is calculated as DTI (%) = [(dual-task feature—single-task feature)/single-task feature] $\times$ 100

## Discussions

In this paper, a specific condition was used to investigate the efficacy of SampEn when a non-unity time delay and multiscale analysis were employed. The specific condition was comparing single and dual-task walking trials in people with PD. Both approaches enhanced the efficacy of single-scale SampEn measure regarding discriminating between single and dual-task trials. Complexity index, which is the summary of multiscale SampEn analysis, was the most effective index of increased risk of fall, showing a significant increase from single-tasking to dual-tasking. Furthermore, while the complexity index of angular velocity signals yielded similar results, they were different from the results of trunk linear acceleration signals. Trunk linear acceleration was the only signal that was able to distinguish between the two walking conditions in three directions (ML, AP, and V).

It has previously been recommended that a time-delay greater than one be incorporated for discrete [19] and continuous time series [48]. However, it was not clear whether SampEn results would be any different if a non-unity time delay was used. In the present study, when SampEn was calculated with a time delay of one, only one out of 24 signals was able to distinguish between the two conditions. This was improved when a non-unity time delay was used, and 6 out of 24 signals showed a significant increase from single to dual-task walking conditions. Interestingly, however, the SampEn of MA-FootAV in the vertical direction, which increased significantly when using a time delay of one, did not change significantly when incorporating a non-unity time delay. Nevertheless, there was an increasing trend from single-task to dual-task. This observation might suggest that the significant difference found for the SampEn of MA-FootAV with a unity time delay is not a true finding and is rather a false positive or a type I error.

Kinematic and kinetic human signals resemble periodic signals with fluctuations from stride to stride. The dependency of the points of kinematic or kinetic signals to their neighboring points might jeopardize the discriminatory ability of the entropy measures. By incorporating a non-unity time delay, templates are constructed such that there is the least dependence between elements of each template [22]. As a result, this preprocessing would help reveal small differences between the two conditions (e.g. single vs. dual tasking) that might have been masked because of the strong periodicity of the signals. In other words, periodic signals result in smaller entropy values compared to non-periodic signals. A non-unity time delay addresses this issue, which yields greater entropy values, and, consequently, increases the discriminatory ability of entropy measures.

Similar to incorporating a non-unity time delay, our study confirms that multiscale SampEn [5] rather than single-scale SampEn can better discriminate between different walking conditions. In addition to a consistent increasing trend from walk only to dual-task walking condition, there were some time scales where values calculated for the dual-task walking condition were significantly larger. The accumulation of these pronounced values resulted in the superior efficacy of the complexity index which is the summary of multi-scale SampEn values. This index was able to successfully detect the effects of dual-tasking using 17 out of 24 signals. The results of our study were similar to the results by Ihlen et al. [3] in terms of the sparsity of significant results in multiscale SampEn plots. Furthermore, in both our study and in the study by Ihlen et al. [3], the direction of change in entropy measure was consistent for all scales. In other words, if one condition resulted in smaller entropy values, this decreasing effect held throughout all time scales.

Each signal in this study was presented in three planes of motion, mediolateral, anteroposterior, and vertical plane. It was hypothesized that the discriminatory ability of SampEn values in the mediolateral direction would be greater using either approach. This was confirmed only for linear acceleration signals obtained from COG and sternum. Bauby and Kuo [49] demonstrated that unlike the AP direction, lower extremity movement in the ML is controlled by higher level cortical structures actively processing visual information. Therefore, the differences in the ML direction for the COG and sternum plausibly arose when visual attention was divided between controlling the upper extremity and performing the visual word searching task. Interestingly, however, no concurrent differences occurred in lower extremity ML motion. In healthy young adults, disruptions to the trunk’s trajectory during walking can be offset by appropriate adaptations in the lower extremity to maintain postural control and dynamic balance [42]. This is primarily due to the fact that the neuromuscular system determines the stepping foot’s final placement (in the AP and ML directions) on its ability to predict the trunk’s future position and velocity [50, 51]. Lack of task differences in our lower extremity ML parameters may suggest a lack of appropriate adaptation in individuals with PD to assess trunk movement changes and alter their base of support [41, 42]. In other words, since COG changes between single and dual-task condition, but there is no parallel change in lower extremity ML parameters, this might suggest that participants were unable to adapt their foot placement to modify their base of support in the ML direction. This may have arisen from differences in task prioritization in people with PD compared to both aged matched controls and young adults [35, 52]. Dual-tasking evidence posits that performance during dual-tasking is not only dictated by two simultaneous tasks competing for attentional resources, but also from an individual’s ability to correctly prioritize the tasks [35]. Indeed, healthy young adults, and to some extent elderly adults, prioritize gait stability (“posture first” strategy) over performance of a secondary cognitive tasks, while people with PD utilize a “posture second” strategy where they prioritize performance of the secondary task over their gait stability [35]. The inability to correctly prioritize gait over the secondary tasks has been associated with deterioration in the prefrontal and anterior cingulate cortices, two higher level cortical structures implicated in the compensation of impaired basal ganglia functionality, in people with PD [35]. However, as this study did not examine differences between people with PD and aged matched controls, the extent to which prioritization affected Entropy measures cannot be elaborated on.

This study investigated the efficacy of SampEn in discriminating between different walking conditions in people with PD when a non-unity time delay and multiscale analysis were used. The results of the complexity index were promising as it was able to highlight small changes made to the signals due to performing a simple secondary task while walking. However, there are a few limitations that need to be considered when interpreting the findings. The secondary task, although being representative of similar situations in every-day life, was not very challenging. 12 out of 17 participants were able to successfully complete the secondary task, i.e. calling out 12 out of 12 words. Moreover, the remaining 5 participants managed to verbally call out the majority of words (mean ± SD, 10 ± 1.5). Additionally, the effect of dual-tasking on entropy measures may vary depending on the relative complexity of the secondary mental task as well as the stage of disease progression in PD.

The question of whether SampEn with and without a non-unity time delay would show more significant results if a more challenging task was used should be investigated in a future study. In addition, although treadmill walking is an efficient and commonly used method to collect data from several strides [41, 53], it may reduce the effect of dual-tasking on gait characteristics [54]. However, it has also been reported that the difference between overground walking and treadmill walking are generally small [53]. Therefore, as the objective of this study was to find the most effective entropy metric rather than generalizing the results to overground walking, this limitation would have a minimal effect on the findings. Finally, performing the secondary task while walking on the treadmill would make participants rotate their head left and right. However, the majority of head rotations were less than 20 degrees as the far left/right displayed words were still in the peripheral vision of our participants. Therefore, they only needed to slightly rotate their head when needed. Nevertheless, not studying the possible effects of this left/right head rotations on the results could be considered as a limitation of this study.

## Conclusions

In summary, the three approaches used in the current study, i.e. single-scale SampEn with and without incorporating a non-unity time delay, multiscale SampEn, and summarizing feature called complexity index, showed different discriminatory performance across 24 continuous signals when comparing single and dual-task walking in people with Parkinson's disease. Incorporating a non-unity time delay improved the discriminatory efficacy of SampEn of 6 signals and the multiscale approach proved that not all scales could distinguish between the two walking conditions. The complexity index of the majority of signals (17 out of 24), however, successfully distinguished normal single-task walking from dual-task walking. Therefore, the complexity index of continuous signals can be used as a gait feature for classifying different treadmill walking conditions.

## Data Availability

The datasets used and analysed during the current study are available from the corresponding author on reasonable request.

## Abbreviations

SampEn: Sample entropy
PD: Parkinson’s disease
MSE: Multiscale sample entropy
CI: Complexity index
ApEn: Approximate entropy
SternumLA: Trunk linear acceleration at sternum level
COG-LA: Center of gravity linear acceleration
FootAV: Foot angular velocity
ShankAV: Shank angular velocity
ThighAV: Thigh angular velocity
ML: Mediolateral
AV: Anteroposterior
V: Vertical
MA: Most affected
LA: Least affected
